# Acetylshikonin inhibits inflammatory responses and Papain-like protease activity in murine model of COVID-19

**DOI:** 10.1038/s41392-022-01220-7

**Published:** 2022-10-27

**Authors:** Ning Lu, Tingxuan Gu, Xueli Tian, Simin Zhao, Guoguo Jin, Fredimoses Mangaladoss, Yan Qiao, Kangdong Liu, Ran Zhao, Zigang Dong

**Affiliations:** 1 Department of Pathophysiology, School of Basic Medical Sciences, Academy of Medical Science, College of Medicine, Zhengzhou University, Zhengzhou, Henan 450001 P.R. China; 2 China-US (Henan) Hormel Cancer Institute, No.127, Dongming Road, Jinshui District, Zhengzhou, Henan 450008 China; 3 Department of Pathology, The Affiliated Cancer Hospital of Zhengzhou University, Zhengzhou, Henan 450008 China; 4 The Henan Luoyang Orthopedic Hospital, Zhengzhou, Henan P.R. China

**Keywords:** Inflammation, Drug screening

**Dear Editor,**

The emerging severe acute respiratory syndrome coronavirus 2 (SARS-CoV-2) variant Omicron has rapidly replaced the Delta variant and presents a huge challenge to public health and health care infrastructure. Cytokine-driven hyperinflammation is the leading cause of a series of COVID-19 clinical symptoms.^1^ Our research attempted to discover an anti-inflammatory or anti-viral small molecule agent capable of treating COVID-19 hyperinflammation.

Papain-like protease (PL pro) is an important protease required for the production of SARS-CoV-2. After the entry of SARS-CoV-2 virus into cells, PL pro participates in the lysis of the SARS-CoV-2 poly-protein and eventually produces non-structural proteins that are essential for viral transcription and replication processes. PL pro also mediates the deubiquitination and deISGylation of antiviral proteins, thus participating in the regulation of host antiviral innate immunity. Therefore, inhibition of PL pro can effectively decrease virus replication. To predict whether a small molecule agent could potentially inhibit SARS-CoV-2 PL pro, we conducted computational molecular docking. The result showed that acetylshikonin could bind to PL pro. As shown, acetylshikonin interacts with PL pro through extensive hydrogen bonding interactions, π-π interactions, and hydrophobic interactions with key residues. Specifically, the acetate group of acetylshikonin forms a hydrogen bond with the guanidyl group of Arg 166, while the hydroxyl group of the naphthalene-1,4-dione is hydrogen bonded with the main chain of Leu 162. In addition, the naphthalene-1,4-dione also forms π-π interactions with Tyr 264, Tyr 268, and Tyr 273. The 2-methylpent-2-ene group forms hydrophobic interactions with the side chains of Tyr 264, Pro 248, and Thr 301 (Fig. 1a).

**Fig. 1:**
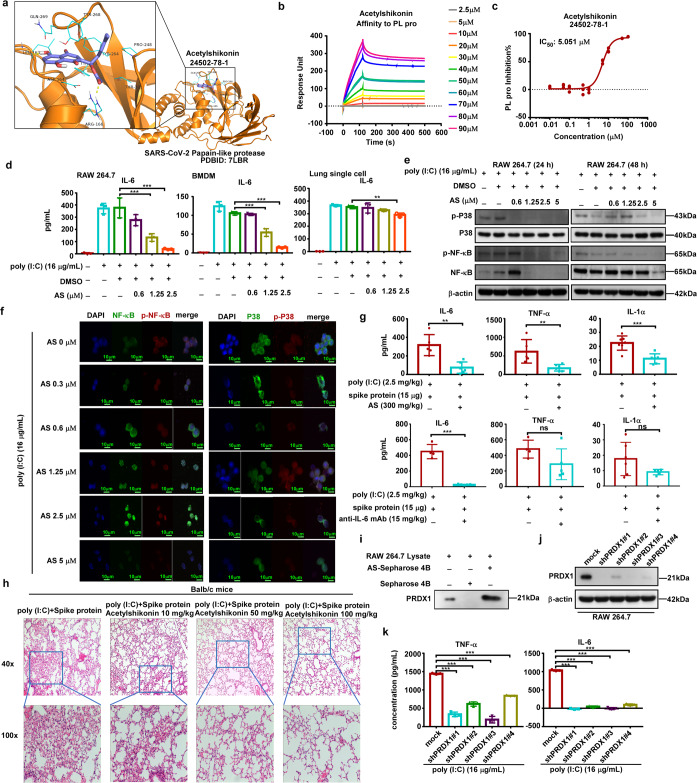
Anti-viral and anti-inflammatory effects of acetylshikonin in vivo and in vitro. **a** The binding mode of acetylshikonin with PL pro (PDBID: 7LBR) predicted by the Autodock VINA program. Acetylshikonin is represented as a purple stick, while PL pro is represented as a ribbon diagram. The key residues interacting with the acetylshikonin are shown in line. **b** The affinity between acetylshikonin and PL pro. **c** Inhibitory rate of different concentrations of acetylshikonin on PL pro. **d** RAW264.7, BMDM and lung single cells were induced with poly (I:C) (16 µg/mL) and treated with different concentrations of acetylshikonin (0, 0.6, 1.25, 2.5 μM) for 12 h. The concentration of IL-6 in the cell supernatant was then quantified by ELISA assay. Data represented the concentration of IL-6. **e** The effect of acetylshikonin on NF-κB signaling in RAW264.7 cells was assessed by Western blot analysis followed by densitometric quantification. The cells were treated with poly (I:C) (16 µg/ml) and 0, 0.6, 1.25 or 5 μM acetylshikonin and harvested at 24 h and 48 h. Cell lysates were then subjected to Western blotting. The results showed the expression of phosphorylated P38 and phosphorylated NF-κB upon treatment with acetylshikonin. **f** Effect on the nuclear translocation of p-NF-κB and p-P38. Cells were treated with poly (I:C) (16 µg/ml) and different concentrations of acetylshikonin. **g** The concentrations of IL-6, TNF-α and IL-1α with 300 mg/kg acetylshikonin and 15 mg/kg anti-IL-6 mAb in an acute lung injury mouse model. **h** H&E staining of lung tissues. (magnification: ×40 m, ×100 m). **i** The binding of acetylshikonin with endogenous PRDX1 present in RAW264.7 cell lysate was determined via ex vivo pull down assay using Sepharose 4B and acetylshikonin-conjugated Sepharose 4B beads. **j** The expression of PRDX1 in RAW264.7 cells expressing shRNA-PRDX1 was evaluated by Western blotting. **k** The concentration of IL-6 and TNF-α with poly (I:C) in RAW264.7 by shRNA silencing of PRDX1. ns. Not significant; *. *P* ≤ 0.05; **. *P* ≤ 0.01; ***. *P* ≤ 0.001

Acetylshikonin is a naphthoquinone derivative mainly extracted from several species of the Boraginaceae family of flowering plants, such as Lithospermum erythrorhizon, and possesses anti-bacterial, anti-inflammatory, anti-viral and anti-cancer effects.^2^ To further confirm the affinity between acetylshikonin and PL pro, we performed the SPR assay using recombinant SARS-CoV-2 PL pro and acetylshikonin. Results indicated that the in vitro interaction between the acetylshikonin and PL pro increased in a dose-dependent manner (Fig. 1b). We next used an in vitro fluorescent peptide substrate to report the activity of PL pro in the presence or absence of acetylshikonin. The results indicated that acetylshikonin could inhibit the protease activity of PL pro; the 50% inhibitory concentration (IC_50_) was calculated as 5.051 µM (Fig. 1c, supplementary Fig. 1a), whereas the IC_50_ was calculated as approximately 23.57 µM for M pro (supplementary Fig. 1b). Deoxyshikonin, an acetylshikonin derivative, was found to inhibit the protease activity of PL pro with an IC_50_ of 7.012 µM (supplementary Fig. 1c). The known PL pro inhibitor, GRL-0617, was used as a positive control; its IC_50_ was measured as 2.731 µM (supplementary Fig. 1d).

To study the anti-inflammatory effect of acetylshikonin, we performed cell-based assays using RAW264.7, BMDM and single cells derived from the lung. Upon poly(I:C) stimulation, IL-6 inflammatory cytokine production increased significantly. We found that acetylshikonin down-regulated the production of IL-6 in a dose-dependent manner in RAW264.7, BMDM and lung single cells. Importantly, IL-6 production decreased significantly at the 2.5 µM acetylshikonin concentration (Fig. 1d) without any alteration in T cell and B cell response (supplementary Fig. 6); there was no significant effect on cell activity within the range of 2.5 µM (supplementary Fig. 2a). To explore the inhibitory mechanism of acetylshikonin, we evaluated the signaling pathways in RAW264.7 cells. The mitogen-activated protein kinase (MAPK) and nuclear factor-κB (NF-κB) signaling pathways were previously reported to be involved in the regulation of inflammatory factors and chemokine expression in macrophages.^3^ Therefore, we detected the levels of phosphorylated p38 and NF-κB in acetylshikonin treated cells. Our results showed that phosphorylated p38 and NF-kB were decreased in a concentration-dependent manner (Fig. 1e). Results obtained from immunofluorescence staining experiments were in agreement with the Western blot experiments; intranuclear p-NF-κB and p-P38 abundance were decreased upon treatment with acetylshikonin in a concentration-dependent manner (Fig. 1f). These findings suggested that acetylshikonin can inhibit inflammatory cytokine production in RAW264.7 cells upon poly (I:C) treatment through regulating the MAPK and NF-kB signaling pathways.

To confirm the anti-inflammatory effects of acetylshikonin in acute lung injury, a COVID-19 related mouse model was used in this study.^4^ Male BALB/c mice were treated via i.g with different concentration acetylshikonin for three consecutive days. The mice were then subsequently stimulated with poly (I:C) and spike protein via intra-trachea injection. Bronchoalveolar Lavage Fluid (BALF) samples were harvested 6 h post challenge and a multi-cytokine assay was performed (supplementary Fig. 3a). The results showed that an anti-IL-6 mAb could significantly decrease IL-6 concentration; however, TNF-α and IL-1α concentrations were not altered. Compared with the anti-IL-6 mAb, the concentrations of IL-6, TNF-α and IL-1α in BALF were significantly decreased after treatment with 300 mg/kg acetylshikonin (Fig. 1g). We then performed an independent animal experiment to explore the minimal dosage of acetylshikonin for prevent acute lung injury. The result indicated that the concentrations of IL-6, and TNF-α in BALF decreased significantly at the 100 mg/kg acetylshikonin concentration; however, no obvious changes in IL-6 or TNF-α production were observed at the 50 mg/kg and 10 mg/kg acetylshikonin concentrations (supplementary Fig. 3b). The H&E staining analysis showed that symptoms of interstitial pneumonia were suppressed in the acute lung injury model upon treatment with acetylshikonin in a concentration-dependent manner (Fig. 1h).

To study the molecular mechanisms contributing to the anti-inflammatory effect of acetylshikonin in the lung, we performed an in-vitro pull-down assay. It has been reported that removal of peroxiredoxin-1 (PRDX1) strongly protected mice from dying of inflammation, and decreased IL-1β, IL-6 and TNF-α productions.^5^ MS assay results showed that acetylshikonin could bind to PRDX1 (supplementary Fig. 4a). Further results indicated that acetylshikonin could selectively bind with PRDX1 in RAW264.7 cell lysate (Fig. 1i).

To verify the role of PRDX1 in the production of inflammatory factors in macrophages, we down-regulated the expression of PRDX1 in RAW264.7 cells by shRNA and detected the production of IL-6 and TNF-α in the culture mediums after stimulation with poly (I:C). We confirmed that silencing of PRDX1 by shRNA abolished the expression of PRDX1 in RAW264.7 cells (Fig. 1j). The results showed that down-regulation of PRDX1 expression significantly reduced the production of the inflammatory cytokines IL-6 and TNF-α induced by poly (I:C) stimulation in RAW264.7 cells (Fig. 1k). However, down-regulation of PRDX1 expression did not significantly reduce the production of inflammatory cytokines IL-6 and TNF-α induced by lipopolysaccharide (LPS) treatment in RAW264.7 cells (supplementary Fig. 5a). Additionally, acetylshikonin treatment significantly decreased TNF-α production in a dose-dependent manner in RAW264.7 cells expressing shRNA-mock (supplementary Fig. 5b) but failed to decrease the TNF-α production in RAW264.7 cells expressing shRNA-PRDX1 (supplementary Fig. 5c). This finding suggests that PRDX1 may be selectively targeted by acetylshikonin.

The rapid spread of SARS-CoV-2 variant of concerns (VOCs) across the globe is responsible for the persistence of the pandemic. Current treatment regimens of COVID-19 include COVID-19 vaccines, mAbs, small molecules and steroids; however, each option has failed to control the increase in infections. Due to the limitations of those therapeutic approaches, we aim to develop a highly efficient drug to control the increasing infections of SARS-CoV-2, provide more chance for those low incoming people and a new strategy to fight with rising infections in SARS-CoV-2. Our study indicated that acetylshikonin could directly bind with PL pro and inhibit its protease activity. We hypothesized it could inhibit the production of inflammatory factors in-vitro and in-vivo through targeting of PRDX1, yet acetylshikonin could not inhibit the production of inflammatory factors in cells depleted of PRDX1 upon stimulation with LPS. This phenotype may indicate differences between viral-infection and bacteria-infection induced inflammatory responses. As acetylshikonin inhibited the inflammatory factors in the mouse model, this compound or its derivatives could be considered for further clinical development of molecular target-based therapy for COVID-19. Moreover, acetylshikonin should be considered for the development of antiviral and anti-inflammatory drugs. Such drugs could be used prophylactically during the COVID-19 window period to prevent mild to severe COVID-19 symptoms.

## Supplementary information

SUPPLEMENTAL MATERIAL

## Data Availability

Data are available on request.
