# Supplementary material for: Acetylshikonin inhibits inflammatory responses and Papain-like protease activity in murine model of COVID-19
Source: Signal Transduct Target Ther. 2022 Oct 27;7:371. doi: 10.1038/s41392-022-01220-7 (PMC9610350; doi:10.1038/s41392-022-01220-7)
Supplement: Supplementary file 1 — SUPPLEMENTAL MATERIAL [file 41392_2022_1220_MOESM1_ESM.docx]

Supplementary Materials for

Acetylshikonin inhibits inflammatory responses and Papain-like protease activity in murine model of COVID-19

Ning Lu^1,2^†, Tingxuan Gu^1,2^†, Xueli Tian^1,2^, Simin Zhao^2,3^, Guoguo Jin^2,4^, Fredimoses Mangaladoss^1,2^, Yan Qiao^1^, Kangdong Liu^1,2^, Ran Zhao^1,2^, and Zigang Dong^1,2^*

^1^Department of Pathophysiology, School of Basic Medical Sciences, Academy of Medical Science, College of Medicine, Zhengzhou University, Zhengzhou, Henan 450001, P.R. China,

^2^China-US (Henan) Hormel Cancer Institute, No.127, Dongming Road, Jinshui District, Zhengzhou, Henan, 450008, China,

^3^Department of Pathology, The Affiliated Cancer Hospital of Zhengzhou University, Zhengzhou, Henan 450008, China,

^4^The Henan Luoyang Orthopedic Hospital, Zhengzhou, Henan, P.R. China

† These authors contributed equally: Ning Lu, Tingxuan Gu

* Correspondence to: Zigang Dong (dongzg@zzu.edu.cn)

This PDF file includes:

Materials and Methods

Supplementary Figures 1 to 6

**Materials and Methods**

**Molecular Docking.** Computational molecular docking of acetylshikonin with PL pro was performed using the Schrodinger Suite software. The PL pro crystal structure was obtained from the Protein Data Bank (PDB ID: 7LBR) and was prepared under the standard procedures of the Protein Preparation Wizard in Schrodinger. Hydrogen atoms were added consistent with pH of 7 and all water molecules were removed. Acetylshikonin was prepared for docking by default parameters using the LigPrep program. Finally, the docking of acetylshikonin with PL pro was performed with the default parameters under the extra precision (XP) mode using the program Glide.

**Cells culture.** All cells were maintained in a humidified incubator under 5% CO_2_ at 37℃. RAW264.7 cells were cultured with the DMEM medium (Vivacell, Cat#2149399) supplemented with 10% FBS (Biological Industries, Cat#2117119), penicillin (100 units/mL), and streptomycin (100 mg/mL). Bone Marrow Derived Macrophages (BMDM) derived from mouse bone marrow were obtained through following method. The mice were sacrificed by cervical dislocation and disinfected by soaking in 75% alcohol. The hind limb humeri of the mice were isolated and washed twice with DPBS. The supernatant was discarded and the humeri were resuspended in RPMI1640 (Biological Industries, Cat#2111071) supplemented with penicillin (100 units/mL), streptomycin (100 mg/mL), 10% FBS (Biological Industries, Cat#2117119) and 50 ng/mL Recombinant Human M-CSF R (Novoprotein, Cat#CP66). The RPMI-1640 medium supplemented with 50 ng/mL M-CSF was changed until cell confluence reached 90%; the BMDM were then used for subsequent experiments. Lung single cells were derived from mouse lung tissue. Male BALB/c mice aged 12 weeks were sacrificed by cervical dislocation and then soaked in 75% alcohol for disinfection. The lung tissue was excised with scissors and then washed with saline. Next, the the lung tissue was cut into pieces and digested with 1 mg/mL collagenase type Ⅰ (Solarbio, Cat#No.C8140) at 37℃. The digested cells were subsequently filtered through a 70 μM cell filter and washed three times with cold PBS. The cells were suspended in DMEM medium supplemented with 10% FBS for subsequent cell culture. Jurkat cells were cultured in RPMI1640 (Biological Industries, Cat#2111071) supplemented with penicillin (100 units/mL), streptomycin (100 mg/mL), and 10% FBS (Biological Industries, Cat#2117119). Human peripheral blood mononuclear cells (PBMCs) derived from COVID-19 vaccinated donor were separated using a ficoll gradient.

**Experimental Animals.** Male BALB/c mice (8-10 weeks) were purchased from Beijing Vital River Laboratory Animal Technology Co., Ltd. (Beijing, China). Mice were kept at a controlled temperature (20±2℃), humidity (50±10%) and a 12 h cycle of light and dark. Mice were provided with adequate food and water. All animal experimental procedures complied with the criteria approved by the Ethics Review Commission of Zhengzhou University (following internationally established guidelines, ID-CUHCI2020001).

**Surface plasmon resonance assay.** We detected the binding affinity between acetylshikonin and PL pro (Sinobiological, Cat#40593-V08E) by SPR assay using a Biacore T-200 (GE Healthcare, UK) with a research-grade CM5 sensor chip (GE Healthcare, UK). The machine was primed with ddH_2_O prior to beginning the experiment. Next, a research-grade CM5 sensor chip was docked into the device and the machine then primed twice with filtered PBS. Next, PL pro was immobilized using an amine-coupling kit (GE Healthcare, UK) according to the Wizard template. Briefly, the surface was first activated with a 1:1 mixture solution of 0.1 M EDC (1-Ethyl-3-(3-dimethylaminopropyl)-carbodiimide hydrochloride) and 0.1 M NHS (N-hydroxy succinimide) at 20 μL/min. Next, 20 μg/mL PL pro diluted in 10 mM sodium acetate (pH 4.5) was immobilized to channel 2 and then reached an expected density of 5000 RU. After immobilization, the channel was blocked with an ethanolamine solution. Channel 1 was left as a reference surface. To evaluate the binding ability, various concentrations of acetylshikonin from 2.5 µM to 90 µM were injected through the two channels at a flow rate of 20 μL/min. The compound was designed for 120 s and 600 s to associate and dissociate, respectively. The surfaces were renewed with a 30 s injection of 10 mM glycine (pH 2.5).

***Ex vivo* binding assay.** RAW264.7 cell lysates (500 μg) or recombinant PRDX1 protein (200 ng) were incubated with acetylshikonin-Sepharose 4B (or Sepharose 4B only as a control) beads in reaction buffer (pH 7.5, 5 mM EDTA, 50 mM Tris–HCl, 1 mM DTT, 150 mM NaCl, 0.2 mM PMSF, 0.01% NP-40, and 20× protease inhibitor) overnight with gentle rotation at 4℃. The next day, the beads were washed three times with washing buffer (pH 7.5, 5 mM EDTA, 50 mM Tris–HCl, 0.01% NP40, 150 mM NaCl, 0.2 mM PMSF and 1 mM DTT). The binding between PRDX1 and beads was visualized by Western blotting.

**FRET assay.** A fluorescence resonance energy transfer (FRET) peptide was designed as a reporter of the activity of SARS-CoV-2 PL pro and M pro. FRET emission of the peptide was measured at 518nm after cleavage by PL pro or M pro. The fluorogenic substrate (5-FAM)-RELNGGAPIK(Dabcyl)S for PL pro and (Dabcyl)-TSAVLQSGFRKMK-(5-FAM) for M pro were synthesized by GenScript. A final 100 nM concentration of substrate per well was mixed with serial diluted acetylshikonin, deoxyshikonin, and GRL-0617 (Topscience, Cat#1093070-16-6) solutions in 10 µL assay buffer (20 mM Tris, pH 7.3). After a short incubation, 90 µL of diluted peptide was added to initiate the reaction. The fluorescence signal a ex/em 485/538 was measured every 2 min for total 120 min with a Thermo Scientific Fluoroskan Ascent instrument.

**shRNA assay.** Each viral vector and packaging vectors (pMD2G, psPAX2, shPRDX1#1–4) were transfected into 293T cells; at ratio of 4:3:1 using the Simple-Fect Transfection Reagent (Signaling Dawn Biotech, Cat#profect-01, Wuhan, Hubei, China) the shRNA oligos are shown below. The virus-enriched medium was harvested 48h post transfection, filtered through a 0.22 μm filter, and stored at −20℃. The cultured RAW264.7 cells were infected using the virus-enriched medium supplemented with 2 μg/ml polybrene (Millipore, Billerica, MA, USA) for 24 h. The next day, the medium was replaced, and the cells were cultured for an additional 48 h. The cells were then selected with puromycin (5 μg/ml) for 48 h. The selected cells were used for subsequent experiments. The RAW264.7 cells were pre-seeded (4×10^4^ cells/well) in 96- well plates for 24 h. The cells were then treated with 16 µg/mL poly (I:C) (poly[I:C]-HMW) (Invivogen, Cat#tlrl-pic) or 1 µg/mL LPS (Invivogen, Cat#tlrl-b5lps) respectively in the 96-well plates for 12 h. The culture supernatant was harvested and store at -20℃ until needed.

F1:5’-CCGGGTGATAGAGCCGATGAATTTACTCGAGTAAATTCATCGGCTCTATCACTTTTTG-3’

R1:5’-AATTCAAAAAGTGATAGAGCCGATGAATTTACTCGAGTAAATTCATCGGCTCTATCAC-3’ (shPRDX1#1);

F2:5’-CCGGCCCATGAACATTCCCTTAATACTCGAGTATTAAGGGAATGTTCATGGGTTTTTG-3’

R2:5’-AATTCAAAAACCCATGAACATTCCCTTAATACTCGAGTATTAAGGGAATGTTCATGGG-3’

(shPRDX1#2)

F3:5’-CCGGCCTTCGACAGATAACAATAAACTCGAGTTTATTGTTATCTGTCGAAGGTTTTTG-3’

R3:5’-AATTCAAAAACCTTCGACAGATAACAATAAACTCGAGTTTATTGTTATCTGTCGAAGG-3’

(shPRDX1#3);

F4:5’-CCGGCGCTCTGTGGATGAGATTATACTCGAGTATAATCTCATCCACAGAGCGTTTTTG-3’

R4:5’-AATTCAAAAACGCTCTGTGGATGAGATTATACTCGAGTATAATCTCATCCACAGAGCG-3’

(shPRDX1#4).

**IF staining.** RAW264.7 cells (2×10^4^ cells/well）were plated in 24-well plate for 24h. Acetylshikonin was added into culture medium at gradient concentrations (0, 0.3, 0.6, 1.25, 2.5, 5µM). The cells were then stimulated with poly (I:C) 16 µg/mL for 24 h. After stimulation, the RAW264.7 cells were harvested and fixed in cold methanol for 15 minutes. Then washed three times with pre-chilled PBS. The TSA Alexa FluorTM 555 Tyramide SuperBoostTM Kit (Invitrogen, Cat#2214476) and TSA Alexa FluorTM 488 Tyramide SuperBoostTM Kit (Invitrogen, Cat#2201632) were used according to the manufacturer’s instructions. Anti-p38 MAPK Antibody (1:40; CST, Cat#9212, USA), anti-NF-κB p65 (D14E12) XP® Rabbit mAb (1:400; CST, Cat#8242, USA), anti-phospho-p38 MAPK (Thr180/Tyr182) (D3F9) XP® Rabbit mAb (1:400; CST, Cat#4511, USA), anti-phospho-NF-κB p65 (Ser536) (93H1) Rabbit mAb (1:1000; CST, Cat#3033, USA) and poly-HRP-conjugated (Invitrogen, Cat#2201632) secondary antibody were incubated. After the reaction was terminated, the nuclei were counterstained with 10 µg/mL DAPI (Solarbio, Cat#No.C0060) and mounted onto glass microscope slides.

**Western blot analysis.** RAW 264.7 cell pellets were lysed on ice for 20 minutes in NP-40 cell lysis buffer (50 mM Tris pH 8.0, 0.5–1% NP-40, 150-mM NaCl, dephosphorylation inhibitor tablets, protease inhibitor cocktail and 1-mM PMSF). After centrifugation at 14,000 g for 20 min, the supernatant was transferred to a fresh tube. Protein concentration was determined by using the BCA Quantification Kit (Solarbio, Cat#PC0020, Beijing, China). The cellular protein extracts were separated by SDS-PAGE and transferred to PVDF membranes. Membranes were blocked using 5% milk in 1× PBS-T (PBS containing 0.05% Tween-20). The membranes were then incubated in anti-Phospho-NF-κB p65 (Ser536) (1:1000, CST, Cat#3033, USA), anti-NF-κB p65 (1:1000, CST, Cat#8242, USA), anti-p38 MAPK (1:1000, CST, Cat#9212, USA), anti-Phospho-p38 MAPK (Thr180/Tyr182) (1:1000, CST, Cat#4511, USA), anti-Prdx1 (1:1000, CST, Cat#8499, USA) or anti-beta-Actin (1:1000, Proteintech, Cat#HRP-60008, Wuhan, China) antibodies for protein visualization via Western blotting. The membranes were washed 3 times in 1×PBST buffer prior to incubation with the appropriate secondary antibodies. The specific proteins were visualized by using ECL.

**Poly (I:C) and spike protein induced lung injury mouse model.** BALB/c mice were used to set up the experimental group and control group respectively. Mice were administered acetylshikonin (10, 50, 100, 300 mg/kg/day) via i.g for three consecutive days and anti-IL-6 mAb (BioX-x-cell, Cat#BE0046) (15mg/kg) before 18 h of challenge, respectively; the control group was administered saline solution. After three days of continuous administration, the mice were anesthetized by intraperitoneal injection of Avertin. The neck muscles and glands of the mice were then stripped to expose the trachea. The trachea was then injected with 2.5 mg/kg poly (I:C) and 15 µg SARS-CoV-2 recombinant Spike protein (ECD-His-tag, Genescript, Cat#Z03481). After 6 h, the mice were anesthetized by intraperitoneal injection of 1% sodium pentobarbital. The trachea was then irrigated three times with 1 mL saline. The first lavage was used to measure cytokines. Blood and lung tissues were harvested for subsequent research.

**Harvest of mouse BALF sample.** The first tube of alveolar lavage fluid obtained from the acute lung injury model induced by poly (I:C) and SARS-CoV-2 spike protein was centrifuged at 2500 rpm for 5min. The supernatant was then collected and stored at -80℃.

**Measure of cytokines in BALF.** Samples of BALF were obtained from the acute lung injury model induced by poly (I:C) and SARS-CoV-2 spike protein. The supernatant was collected by centrifugation, and stored at -80℃, as described earlier. Next, the concentration of various cytokines in the BALF, such as IL-6 and TNF-α, IL-1α were measured using the LEGENDplex™ Mouse Inflammation Panel (Biolegend, Cat#740446). The data were collected by flow cytometry using FACSCalibur (BD).

**ELISA assay.** RAW264.7 cells, BMDM cells and single lung cells were seeded into 12-well plates at a density of 6×10^5^ cells/well for 24 h. The cells were treated with various concentrations of acetylshikonin and 16 µg/mL poly (I:C). DMEM with and without poly (I:C) was used as positive and negative controls, respectively. The cells were then incubated for 12 h at 37℃. The supernatant was collected and stored at -20℃ until needed. Next, the Mouse IL-6 ELISA Kit (MULTI SCIENCES, Cat#EK206/3-96) and the Mouse TNF-α ELISA KIT (MULTI SCIENCES, Cat#EK282/4-96) were used to detect and analyze the concentration of IL-6 and TNF-α , respectively. Jurkat cells (4×10^4^ cells/well) were pre-seeded into 96-well plates for 24h. Afterward, the cells were stimulated with PMA (25 ng/mL) and ionomycin (1μg/mL); the RPMI-1640 medium with and without PMA (25 ng/mL) and ionomycin (1μg/mL) were used as positive and negative controls. Various concentrations of acetylshikonin were added into culture medium and then incubated in a 37℃ incubator for 12 h. The cell culture medium was then harvested and stored at -20℃ until needed. Next, IFN-gamma production was detected and analyzed using the Human Interferon Gamma/IFN gamma/IFNG ELISA Kit (Sino Biological, Cat#KIT11725A). Human peripheral blood mononuclear cells (PBMCs) from healthy COVID-19 vaccinated donor (4×10^4^ cells/well) were seeded into 96-well plates with SARS-CoV-2 spike protein (0.1mg/mL) (GenScript, Cat#Z03481-1) and various concentrations of acetylshikonin; the cells were incubated for 48 h at 37℃. RPMI-1640 medium with and without SARS-CoV-2 spike protein (0.1mg/mL) were used as positive and negative controls, respectively. The supernatant was then harvested and stored at -20℃ until needed. Next, SARS-CoV-2 IgG production was detected and analyzed using SARS-CoV-2 RBD Antibody (IgG) ELISA Kit (Vazyme, Cat#DD3112) according to the manufacturer’s instructions.

**Histological Examination.** Whole lungs excised from the acute lung injury mouse models were fixed in 4% paraformaldehyde for approximately 24h, cleaned and embedded in paraffin. 3-4 mm slices were sectioned, stained with hematoxylin and eosin (H&E), and photographed for analysis.

**Cell viability assay.** RAW264.7 cells, BMDM cells and lung single cells were pre-seeded into 96-well plates at a density 4×10^4^ cells/well and allowed to settle for 24 hours. Various concentrations of acetylshikonin (0, 0.6, 1.25, 2.5 µM) were added to labeled wells. 20 µL CCK-8 (Meilunbio, Cat#MA0218-3-Mar-27G) was added to each well after 4, 8, and 12 hours. The absorbance of each well at the indicated time point was measured at 450 nm using the Thermo Multiskan plate-reader (Thermo Fisher Scientific, Waltham, MA, USA).

**Statistical Analysis.** GraphPad Prism 7.0 (GraphPad Software, United States) was used to conduct statistical analysis. Comparison between multiple groups were analyzed using one-way ANOVA, with a Turkey post-hoc. P < 0.05 was considered statistically significant; the data was shown as mean ± SEM.

**Illustrations.** Representative cartoon panels for all figures were generated using BioRender (https://biorender.com/).

**Supplementary Figures**


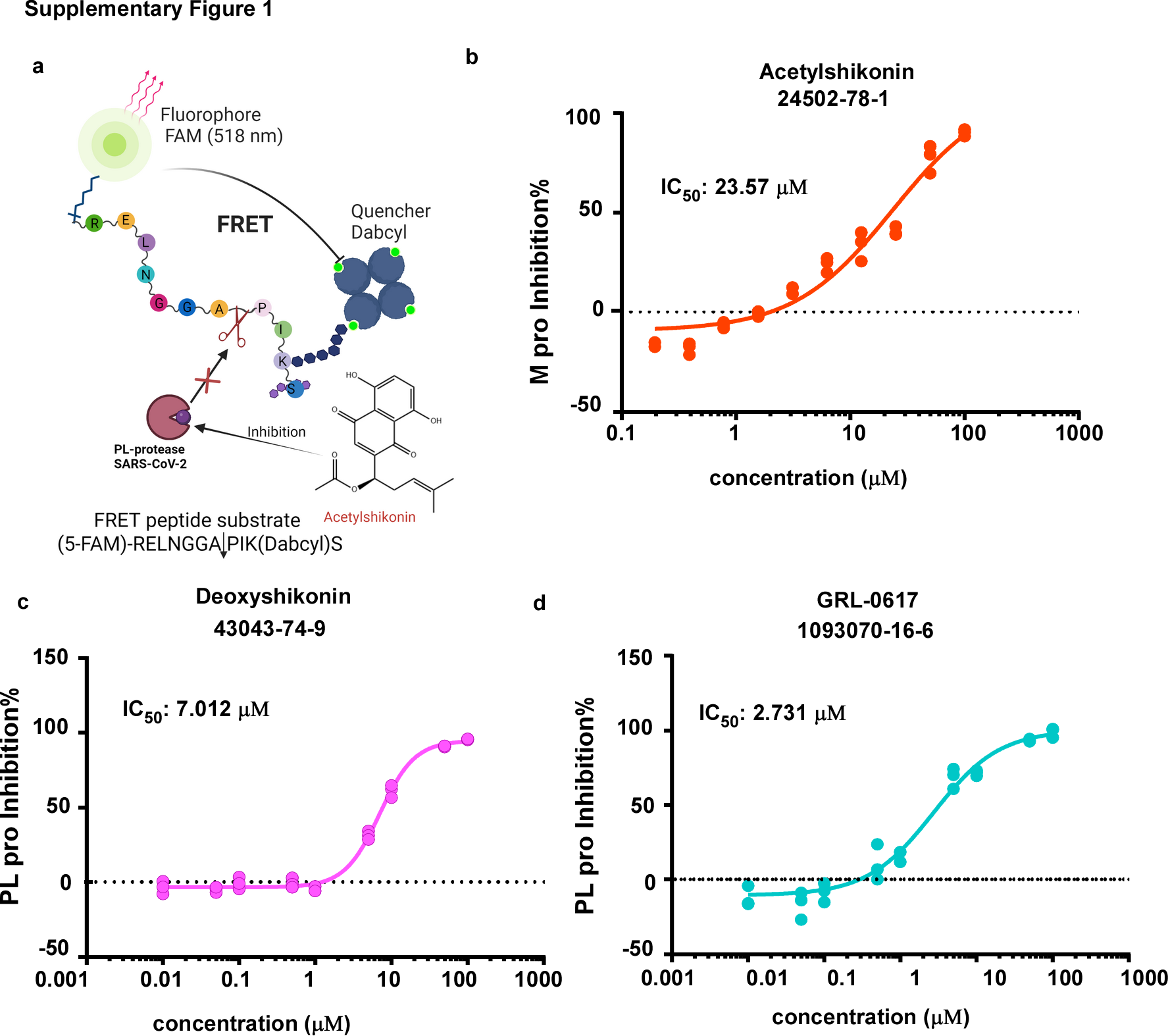


**Supplementary Figure 1. Acetylshikonin exerts anti-PL pro effects in vitro.**

(**a**) Schema of the FRET assay used to visualize the interaction between acetylshikonin and PL pro. Created with Biorender.com. (**b**) Inhibitory rate of different concentrations of acetylshikonin on M pro. (**c**) Inhibitory rate of different concentrations of deoxyshikonin on PL pro. (**d**) Inhibitory rate of different concentrations of GRL-0617 on PL pro.


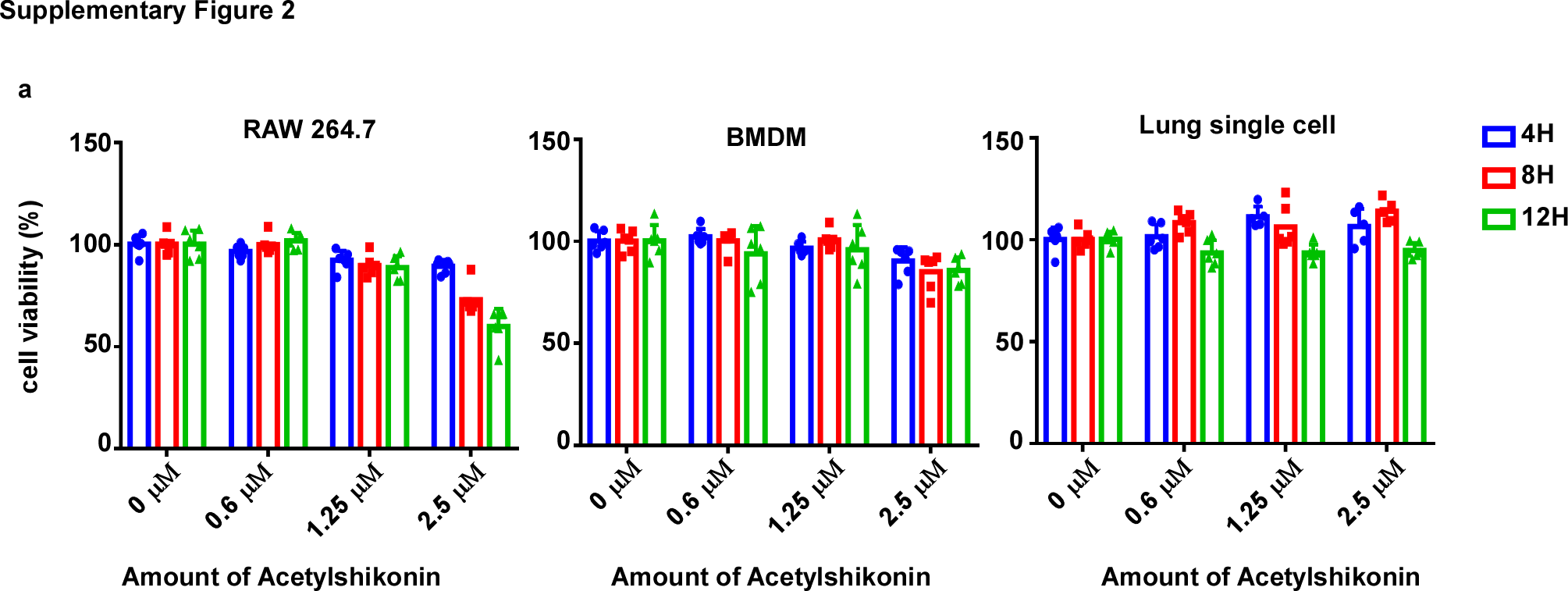


**Supplementary Figure 2. The toxicity of acetylshikonin was determined by cell viability assay.**

(**a**) Cytotoxicity of acetylshikonin was determined using a CCK-8 assay. RAW 264.7, BMDM and lung single cells were treated with the indicated concentrations of acetylshikonin for 4, 8, 12 h.

**
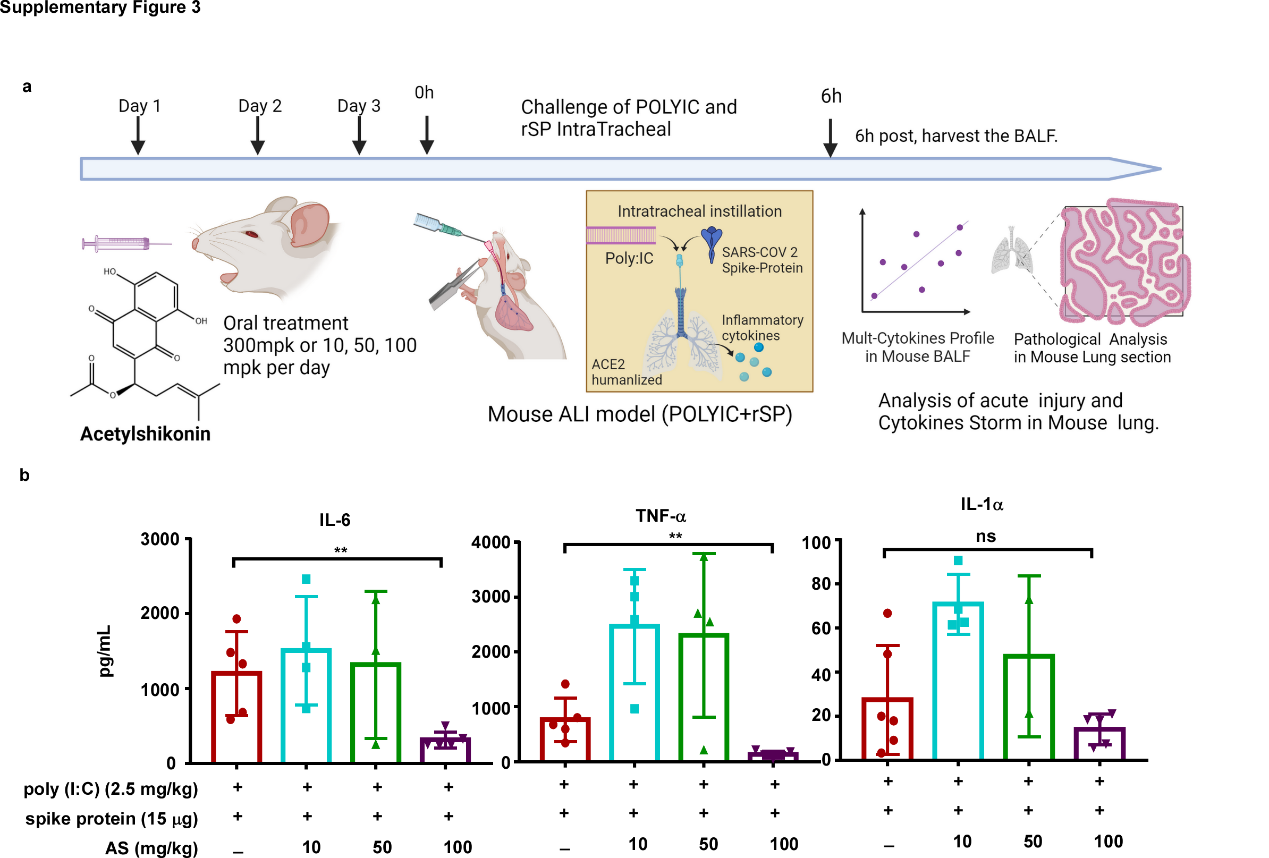
**

**Supplementary Figure 3. Acetylshikonin exerts anti-inflammatory effects in an acute lung injury mouse model.**

(**a**) Schema illustrating the establishment of the acute lung injury mouse model. Created with Biorender.com. (**b**) The concentration of IL-6, TNF-α and IL-1α with 10, 50, 100 mg/kg acetylshikonin in acute lung injury model. (n=6), **p < 0.01; n.s. Not significant.


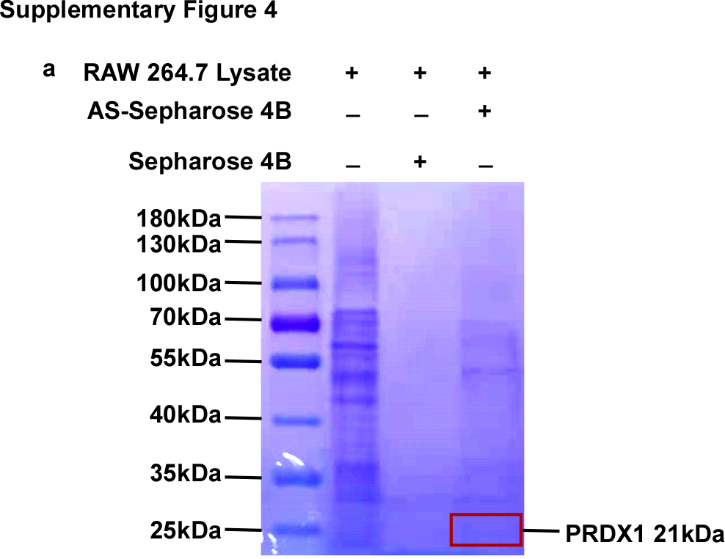


**Supplementary Figure 4.** **Target protein identification by pull-down MS assay.**

(**a**) The binding of acetylshikonin with endogenous PRDX1 present in RAW264.7 cell lysate was determined using Sepharose 4B and acetylshikonin-conjugated Sepharose 4B beads by MS assay.


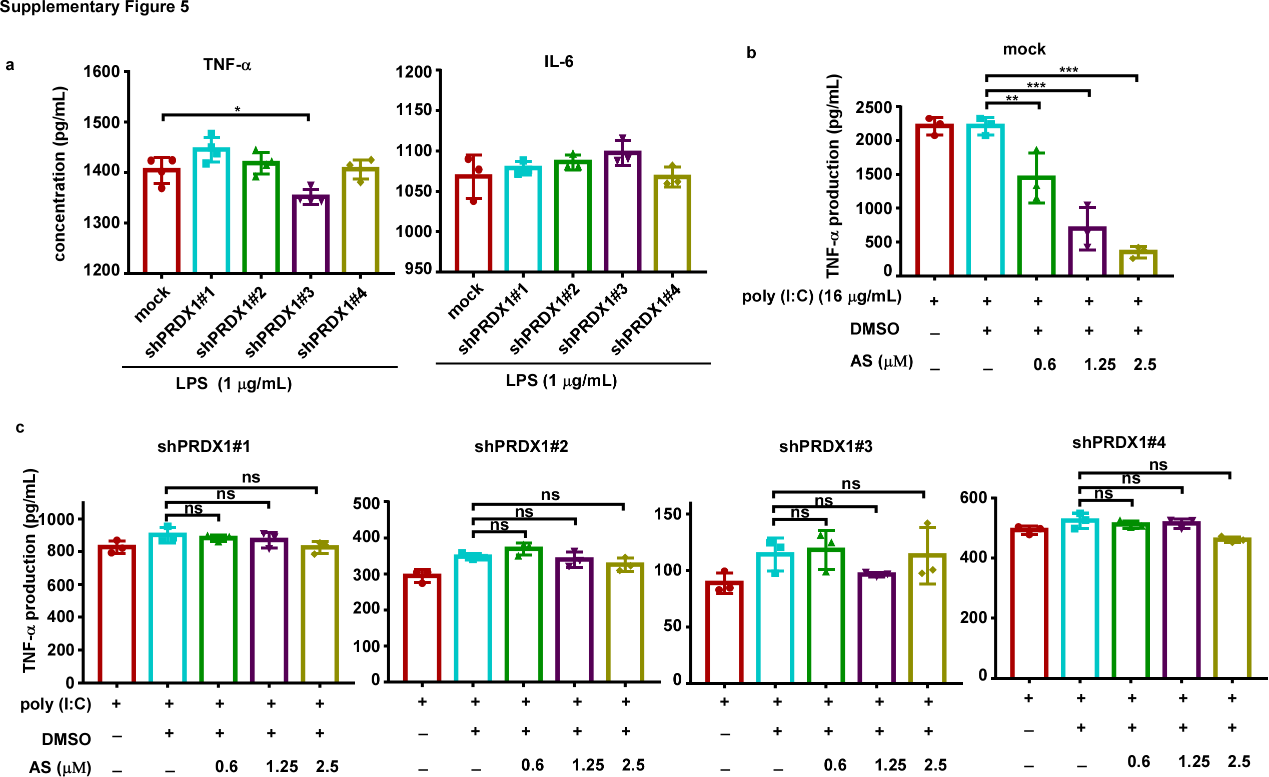


**Supplementary Figure 5. PRDX1 is a potential target of acetylshikonin in RAW 264.7 cells.**

(**a**) The concentration of IL-6 and TNF-α with LPS (1 µg/mL) stimulation in RAW264.7 was measured after shRNA silencing of PRDX1 expression. (**b**) RAW264.7 expressing shRNA-mock were stimulated with poly (I:C) (16 µg/mL) and incubated with various concentrations of acetylshikonin (0, 0.6, 1.25, 2.5 μM) for 12h; TNF-α concentration in the culture medium was subsequently measured. (**c**) RAW264.7 expressing shRNA-PRDX1 were stimulated with poly (I:C) (16 µg/mL) and incubated with various concentrations of acetylshikonin (0, 0.6, 1.25, 2.5 μM) for 12h; TNF-α concentration in the culture medium was subsequently measured. ns. Not significant; *. P ≤ 0.05; **. P ≤ 0.01; ***. P ≤ 0.001.


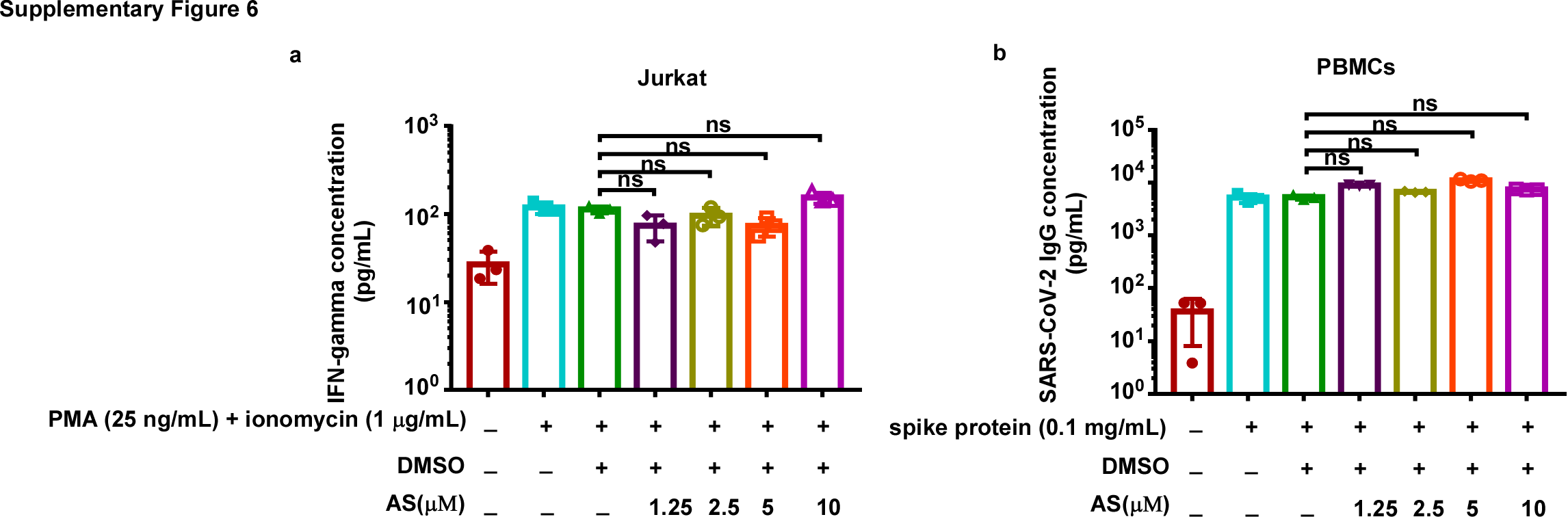


**Supplementary Figure 6.** **Acetylshikonin exerts anti-viral and anti-inflammatory effects** **without altering T cell and B cell response.**

(**a**) IFN-gamma production by Jurkat cells was measured after 12 h incubation with PMA (25 ng/mL) + ionomycin (1μg/mL) and the indicated concentrations of acetylshikonin. (**b**) SARS-CoV-2 IgG production by PBMCs was measured after 48 h incubation with spike protein (0.1mg/mL) and the indicated concentrations of acetylshikonin. ns. Not significant.
